# The survival of epidemic and sporadic MRSA on human skin mimics is determined by both host and bacterial factors

**DOI:** 10.1017/S0950268822001765

**Published:** 2022-11-16

**Authors:** Valérie O. Baede, Michella M. Voet, Tanny J. K. van der Reijden, Annelies van Wengen, Deborah E. Horst-Kreft, Nicole A. Lemmens-den Toom, Mehri Tavakol, Margreet C. Vos, Peter H. Nibbering, Willem J. B. van Wamel

**Affiliations:** 1 Department of Medical Microbiology and Infectious Diseases, Erasmus MC University Medical Center Rotterdam, Rotterdam, the Netherlands; 2 Department of Infectious Diseases, Leiden University Medical Center, Leiden, The Netherlands

**Keywords:** Bacterial infection, epidermal model, innate immunity, keratinocytes, MRSA, virulence factors

## Abstract

Bacterial survival on, and interactions with, human skin may explain the epidemiological success of MRSA strains. We evaluated the bacterial counts for 27 epidemic and 31 sporadic MRSA strains on 3D epidermal models based on N/TERT cells (NEMs) after 1, 2 and 8 days. In addition, the expression of antimicrobial peptides (hBD-2, RNase 7), inflammatory cytokines (IL-1*β*, IL-6) and chemokine IL-8 by NEMs was assessed using immunoassays and the expression of 43 *S. aureus* virulence factors was determined by a multiplex competitive Luminex assay. To explore donor variation, bacterial counts for five epidemic and seven sporadic MRSA strains were determined on 3D primary keratinocyte models (LEMs) from three human donors. Bacterial survival was comparable on NEMs between the two groups, but on LEMs, sporadic strains showed significantly lower survival numbers compared to epidemic strains. Both groups triggered the expression of immune factors. Upon interaction with NEMs, only the epidemic MRSA strains expressed pore-forming toxins, including alpha-hemolysin (Hla), gamma-hemolysin (HlgB), Panton-Valentine leucocidin (LukS) and LukED. Together, these data indicate that the outcome of the interaction between MRSA and human skin mimics, depends on the unique combination of bacterial strain and host factors.

## Introduction

A large part of the general population is a persistent or intermittent carrier of *Staphylococcus aureus* [1]. *S. aureus* is an opportunistic pathogen and carriage increases the risk of subsequent infection [1] ranging from skin and soft-tissue infections (SSTI) to invasive life-threatening infections, such as endocarditis and sepsis [1]. Treatment of these infections is hindered by the emergence of methicillin-resistant *S. aureus* (MRSA), and especially community-acquired (CA)-MRSA which has become a significant cause of SSTI worldwide [2].

MRSA emerged originally as a limited number of clonal complexes (CCs), and these still represent the global dominant clusters today [3, 4]. Some examples are ST5-MRSA-II (CC5), ST239-MRSA-III (CC8) and ST8-MRSA-IV (CC8), also known as USA300 [2, 4]. On occasion, dominant clusters are replaced by others. In the United Kingdom, a shift in dominant MRSA clusters occurred between 2001 and 2007, from EMRSA-16 (CC30) to EMRSA-15 (CC22) [5]. Likewise in North America, USA300 replaced the former dominant USA400 (CC1) and became the major cause of SSTI within five years after its emergence [2, 6].

While epidemic strains become dominant due to successful transmission, sporadic strains fail to disseminate widely despite similar geographical and societal circumstances for transmission. The epidemiological success of USA300 has been studied extensively. Suggested success factors are increased virulence through the production of toxins, such as alpha-toxin and Panton-Valentine leucocidin (PVL), the uptake of the arginine catabolic mobile element, and the acquisition of fluoroquinolone resistance [6–9]. However, while these factors may have advanced transmission of USA300, they do not fully explain its success [6].

In addition to increased virulence, host-pathogen interactions may have influenced the epidemiological success of MRSA strains. Since *S. aureus* carriage on skin increases the risk of subsequent infection and transmission to others, its ability to survive on skin forms the basis of successful transmission [1, 10]. Nevertheless, it remains unknown whether epidemic MRSA are more capable of survival on the skin in comparison to sporadic MRSA.

The aim of this study was to compare the ability of epidemic *vs.* sporadic MRSA strains to survive on human skin. First, human epidermal models based on N/TERT keratinocytes were exposed to 27 epidemic and 31 sporadic MRSA strains. Viable bacteria were quantified and the expression of 5 host immune factors and 43 bacterial virulence factors was determined. Bacterial presence in the culture subnatants was determined to assess the ability of the strains to breach the epidermal barrier function. Finally, epidermal models based on keratinocytes from different human donors were exposed to five epidemic and seven sporadic MRSA strains to explore potential skin donor variation.

## Methods

### Mrsa strains

MRSA strains originated from the MACOTRA strain collection as assembled by the MACOTRA study group to investigate transmission success of MRSA [11]. This collection contains clinical MRSA strains of British, French and Dutch origin, labelled as epidemic or sporadic based on country-specific definitions. In total, 27 epidemic and 31 sporadic MRSA strains of 8 different genetic lineages were included in this study, comprising 16 representatives from France, 26 from the Netherlands and 16 from the UK (Supplementary Table S1). Twelve strains were selected to study the production of bacterial virulence factors and for additional experiments on Leiden epidermal models (LEMs). For each country, four strains were included in this subset, consisting of five epidemic and seven sporadic representatives (Supplementary Table S1). For each batch of epidermal models, MRSA strains LUH14616 (ST247; NCCB100829) and LUH15091 (ST121) were included as bacterial growth controls with phosphate buffered saline (PBS) as negative control [12, 13]. Strains were cultured on Trypticase Soy Agar supplemented with 5% sheep blood (Becton Dickinson, Vianen, The Netherlands) at 37 °C overnight.

### Epidermal model construction

N/TERT-based epidermal models (NEMs) were constructed as described with minor modifications [14]. In short, human keratinocytes of the N/TERT cell line (Harvard Medical School [15]) were cultured to 80% confluency in Keratinocyte Serum Free Medium with L-glutamine (Gibco, Paisley, Scotland) supplemented with 25 μg/ml bovine pituitary extract, 0.2 ng/ml recombinant human epidermal growth factor 1–53, 0.3 mM CaCl_2_ and penicillin-streptomycin at 37 °C and 5% CO_2_. To construct epidermal models, 2 × 10^5^ N/TERT keratinocytes in DermaLife K Keratinocyte Complete Medium with LIFE factors (LifeLine Cell Technology, Frederick, USA) were seeded onto cell culture filter inserts with 0.4 μm pores (ThinCerts; Greiner Bio-One, Frickenhausen, Germany) in 12-well plates. The cells were cultured 3 days until confluency followed by replacement of the apical and basal medium for DMEM/Ham's F-12/CnT-Prime 3D barrier medium in a 3:1:4 ratio supplemented with 0.1 μg/ml hydrocortisone, 0.125 μg/ml isoprotenerol, 0.25 μg/ml bovine insulin, 16.5 pM selenious acid, 5 mM L-serine, 5 μM L-carnitine, 1.6 mg/ml BSA, 25 μM palmitic acid, 15 μM linoleic acid and 7 μM arachidonic acid. The apical medium was removed after 24 h, thereby leaving the top cell layers air-exposed and allowing epidermal differentiation. The basal medium was replaced after 3 days of air exposure to the same medium with an increased concentration of linoleic acid (30 μM). This medium was refreshed every 2 days. After 10 days of air-exposure, NEMs were fully differentiated and ready for experimentation.

LEMs were based on primary keratinocytes originating from 3 different donors. For our study, fresh plastic surgery surplus skin samples were collected from patients after informed consent. This procedure is in accordance with the Dutch Law and additional approval from ethics committee was not required. According to Article 467 of the Dutch Law on Medical Treatment Agreement and the Code for proper Use of Human Tissue of the Dutch Federation of Biomedical Scientific Societies, coded anonymous surplus tissue can be used for biomedical research when the donor has no objection [16]. The Helsinki Declaration principles were followed while working with human tissue.

For keratinocyte isolation, the epidermis of fresh surplus skin was mechanically separated from the dermis and digested to obtain a keratinocyte cell suspension. Next, keratinocytes were cultured in Keratinocyte medium, comprising of DMEM and Ham's F-12 medium at 3:1 ratio supplemented with 5% foetal bovine serum, 0.5 μM hydrocortisone, 1 μM isoproterenol, 0.1 μM insulin, 100 U/ml penicillin and 100 μg/ml streptomycin. Keratinocyte medium was switched to DermaLife with 100 U/ml penicillin and 100 μg/ml streptomycin before the construction of the 3D models. After 24 h, 2 × 10^5^ keratinocytes were seeded onto filter inserts (0.4 μm Costar inserts) in 24-wells plates in DermaLife medium. After 3 days, keratinocytes were air-exposed by removing the apical medium. The basal medium was replaced with CnT-02-3D medium and keratinocyte medium supplemented with 240 nM BSA, 25 μM palmitic acid, 15 μM linoleic acid and 7 μM arachidonic acid. Experiments were performed using 10 days air-exposed cultures.

### Epidermal model colonisation

Before bacterial inoculation, the model medium was replaced by fresh medium without antibiotics. After 24 h, models were inoculated with 1 × 10^5^ CFU/ml log phase MRSA in PBS. After 1 h at 37 °C in 5% CO_2_, non-adherent bacteria were plated and counted. At 24 h, 48 h and 8 days (NEMs) after inoculation, the subnatant, the cell culture medium beneath the air-exposed keratinocyte layer, was screened for bacterial contamination, the models were washed, and the non-adherent bacterial counts were determined. The models were homogenised using a Precellys 24 tissue homogenizer (3 × 10s at 5000 rpm, Bertin Technologies, Montigny-le-Bretonneux, France) and adherent bacterial counts determined. The lower detection limit for adherent bacteria was 40 CFU/model. For each strain, six replicates of NEMs were prepared, and LUH14616 and LUH15091 served as growth controls; NEMs were incubated with PBS as negative controls.

Bacterial counts on LEMs after 24 h and 48 h were evaluated for 12 strains in duplicate as described above. Due to limited donor material, each strain was tested on epidermal models of two different donors. LUH14616 was included as a growth control on models of all three donors, and incubated with PBS as negative controls.

### Production of antimicrobial peptides, cytokines and chemokines by keratinocytes

NEM subnatants were collected at 24 h and 48 h after the start of the infection. Duplicates were pooled and subsequently stored at −20 °C until further analysis. The levels of antimicrobial peptides human *β*-defensin (hBD)-2 (LSBIO) and RNase 7 (DS Develop), cytokines interleukin-1*β* and interleukin-6 and chemokine interleukin-8 (CXCL-8) (all Biolegend) were determined by enzyme-linked immunosorbent assay according to the manufacturer's instructions. The lower limits of detection of hBD-2, RNase 7, IL-1*β*, IL-6 and IL-8 were 12 pg/ml, 0.61 ng/ml, 0.5 pg/ml, 15.6 pg/ml and 7.8 pg/ml, respectively.

### Bacterial virulence factor expression

The presence of 43 bacterial proteins was detected by a multiplex competitive Luminex assay as described, with slight modifications [13, 17]. Proteins of interest were covalently coupled to the beads as previously described [18–20]. The tested proteins include (pore-forming) toxins, exfoliative toxins, immune evasion proteins, staphylococcal enterotoxins, staphylococcal superantigen-like proteins, MSCRAMMs (microbial surface components recognising adhesive matrix molecules) and others. An overview of included proteins is listed in Supplementary Table S2.

For a subset of 12 MRSA strains, five three-fold serial dilutions of pooled NEM subnatant duplicates, collected at 24 h, were made in PBS supplemented with 1% (w/v) bovine serum albumin (Roche Diagnostics, Almere, Nederland) and 0.05% (w/v) sodium azide (Sigma-Aldrich, Zwijndrecht, Nederland). These were incubated in equal volumes with a 1:50 dilution of polyclonal human IgG (Sigma-Aldrich) for 35 min at 25 °C with continuous shaking at 800 rpm. Incubated subnatant was added in equal volumes to a prepared black flat-bottomed 96-well plate containing 50 μl of bead mixture. Mean Fluorescent Intensity (MFI) of IgG-bound beads was measured using a multiplex bead-based flow cytometry (Bio-Plex 200, Bio-Rad, Lunteren, Nederland). An uncoupled bead was used as an internal negative control. As experimental controls, subnatants of bacterial growth controls and PBS-treated models were included in the analysis. To control for bacterial interaction with cell culture medium and filter inserts, a separate experiment was carried out where bacteria were inoculated directly onto the cell culture filter without keratinocytes, inserted into the same basal medium as described above for NEMs.

MFI values were plotted to evaluate the levels of captured IgG throughout the subnatant dilution series. Decrease of MFI values was most pronounced in the first three-fold dilution, which was selected for further analysis. Proteins with values <100 MFI were excluded for data analysis. For the remaining proteins, the relative decrease of MFI in comparison to the untreated control (set at 100% MFI) was calculated. This relative MFI decrease forms a semi-quantitative measure which reflects the presence of the bacterial virulence factor in the subnatant. For each combination of strain and bacterial protein, the difference in relative MFI decrease was calculated between models with and without keratinocytes. If this difference was >20, protein expression was attributed as due to interaction with keratinocytes. A relevant level of protein expression was determined at a cut-off value of 25% in relative MFI decrease.

### Statistical analysis

Before statistical testing, descriptive statistics, histograms and QQ plots were evaluated. Results of non-normal distributed data are given as median and range. Statistical analyses were performed using non-parametric tests, i.e., Wilcoxon's rank sum test for independent samples, Wilcoxon signed rank test to account for paired samples or Kruskal Wallis rank sum test for multiple groups. Statistical significance was set at *α* = 0.05, and all analyses were carried out using R *stats* and *pastecs* packages [21, 22]. All figures were made using the *ggplot2* and *patchwork* packages [23, 24].

### Data availability statement

The authors declare that all relevant data of this study are available within the article or from the corresponding author on reasonable request.

## Results

### Bacterial survival on N/TERT epidermal models (NEMs)

**Fig. 1.**
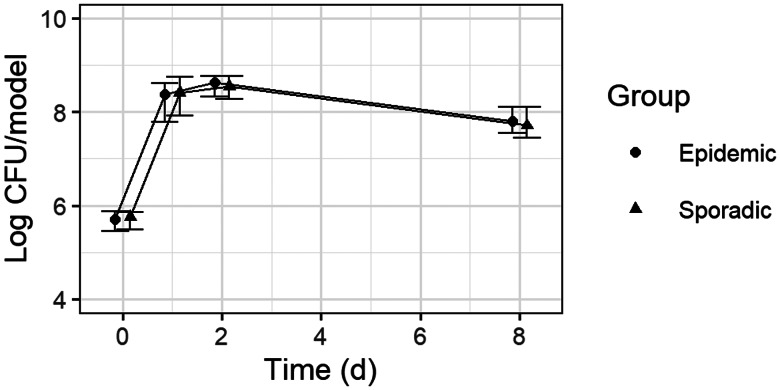
**Course of bacterial counts on N/TERT epidermal models.** Bacterial counts for 27 epidemic and 31 sporadic MRSA strains on NEMs were determined at 24 h, 48 h and 8 days after inoculation of the models; each strain was tested in duplicate. Median log CFU/NEM is shown for epidemic and sporadic strains, with error bars depicting upper and lower quartiles.

To compare the ability of 27 epidemic and 31 sporadic MRSA strains to survive on human skin, NEMs were inoculated, and bacterial counts of viable adherent and non-adherent MRSA were determined after 24 h, 48 h and 8 days. Median log CFU/NEM at inoculation was 5.72 (4.00–6.10). Bacterial numbers increased up to 48 h after inoculation (8.60 log CFU/NEM (6.02–9.31)) and subsequently decreased to 7.77 log CFU/NEM (6.02–8.83). No difference in counts on NEMs was found between epidemic and sporadic strains for 8 days. For example, the median log CFU/NEM was 7.80 (6.10–8.83) for epidemic strains, and 7.71 (6.02–8.59) for sporadic strains at 8 days (*W* = 6421.5; *P* = 0.29) (Fig. 1).

### Bacterial survival on epidermal models from 3 different human donors

**Fig. 2.**
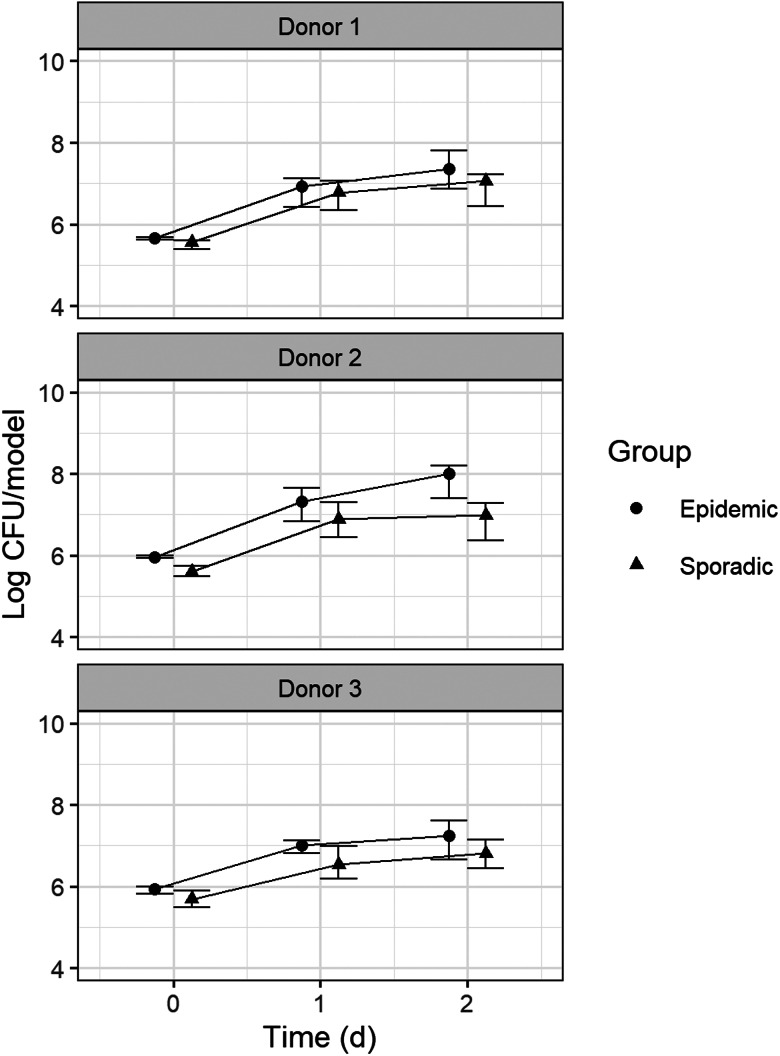
**Bacterial counts on Leiden epidermal models (LEMs) from 3 different human donors.** Bacterial counts for 5 epidemic and 7 sporadic MRSA strains on LEMs from 3 different donors were assessed after 24 h and 48 h. Each strain was tested on LEMs from 2 different donors (due to limited supply), in duplicate. Four epidemic strains and six sporadic strains were tested on LEMs from donor 1. Three epidemic and four sporadic strains were tested on LEMs from donor 2. Three epidemic and four sporadic strains were tested on LEMs from donor 3. Median log CFU/NEM is shown for epidemic and sporadic strains on each donor, with error bars depicting upper and lower quartiles.

To explore potential differences in survival due to host variation, five epidemic and seven sporadic MRSA strains were tested on LEMs based on primary keratinocytes from three donors. Inoculation numbers were comparable for LEMs of the three different donors (5.69 log CFU/NEM (5.37–6.00)). These numbers increased to an overall 7.11 log CFU/LEM (4.46–8.37) over 48 h. Sporadic strains showed slightly lower survival numbers compared to epidemic strains at 24 h and 48 h on LEMs of all donors (Fig. 2). These small differences were significant (*W* = 1192, *P* = 0.03 at 24 h; *W* = 1400, *P* = 0.003 at 48 h). Differences in bacterial counts for sporadic *vs.* epidemic strains were most pronounced on LEMs of donor 2 at 48 h (*W* = 109, *P* = 0.02).

### Production of inflammatory cytokines/chemokines and antimicrobial peptides by N/TERT epidermal models in response to epidemic and sporadic MRSA

**Fig. 3.**
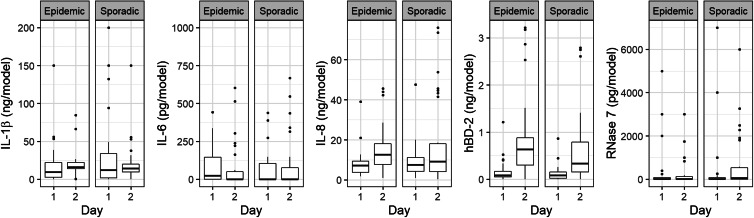
**Production of inflammatory cytokines/chemokines and antimicrobial peptides by N/TERT epidermal models in response to epidemic and sporadic MRSA.** IL-1*β*, IL-6, chemokine IL-8 and antimicrobial peptides hBD-2 and RNase 7 production by keratinocytes at 1 and 2 days after inoculation with 27 epidemic and 31 sporadic MRSA strains. Boxplots (in the style of Tukey) show median values with upper and lower hinges corresponding to the first and third quartiles and whiskers representing ± 1.5 * IQR.

To study the response of N/TERT epidermal models to bacterial exposure, the levels of cytokines IL-1*β* and IL-6 and chemokine IL-8 and of the antimicrobial peptides hBD-2 and RNase 7 in NEM subnatants were determined for all 58 strains at 24 h and 48 h after inoculation of the models. Median values of hBD-2 (*V* = 90, *P* < 0.001), RNase 7 (*V* = 286, *P* > 0.05), IL-1*β* (*V* = 749, *P* > 0.05) and IL-8 (CXCL-8) (*V* = 413, *P* < 0.001) increased during infection, but not for IL-6 (Fig. 3). No differences were found in production of these immune factors between NEMs upon exposure to epidemic or sporadic MRSA strains.

### Production of immune modulators, toxins and other proteins by sporadic and epidemic MRSA strains during infection of N/TERT epidermal models

For a subset of 12 strains, the bacterial response upon exposure to N/TERT cells was studied by determining the presence of 43 bacterial proteins in NEM subnatants at 24 h after inoculation by multiplex competitive Luminex assay. For 11 of 12 MRSA strains, relevant protein expression attributed to interaction with keratinocytes was found (Table 1). Autolysin Atl2 (11 of 12 strains) was most expressed, followed by gamma-hemolysin B (HlgB; 6/12), alpha-toxin (Hla; 5/12), leukocidins LukD (4/12), LukE (2/12) and LukS (2/12), autolysin Aly (2/12), extracellular adherence protein Eap (2/12) and lipase (2/12). For thermonuclease (nuc), staphylococcal enterotoxin-like O (SEO), staphylococcal superantigen-like protein 5 (SSL5), SSL9 and toxic shock syndrome toxin-1 (TSST-1) expression was found in one strain. Aly, Eap, Hla, LukD, LukE and LukS expression was restricted to epidemic strains only. HlgB was expressed in all 5 epidemic strains and 1 sporadic strain. Due to the low MFI values, data for bacterial proteins Esx-1-associated factor EsxB, Staphylococcal enterotoxin E (SEE) and hypothetical protein SA2097 were excluded from the analysis.

**Table 1.** Presence of bacterial virulence factors in N/TERT epidermal model subnatants at 24 h after inoculation.

Strain	Genetic background	Infection or carriage	Epidemic or sporadic	Filter passage at 24 h	Aly^1^	Atl2^1^	Eap^1^	Hla^1^	HlgB^1^	Lipase^1^	LukD^1^	LukE^1^	LukS^1^	Nuc^1^	SEO^1^	SSL5^1^	SSL9^1^	TSST-1^1^
11002	CC8	Carriage	Epidemic	0%		93.7		86.9	61.0		42.8		88.5					
11016	CC5	Carriage	Epidemic	100%	94.8	94.9		71.4	74.6	89.2	35.6							
11127	CC22	Infection	Epidemic	0%		95.5	38.5	82.1	56.9									
11257	CC5	Infection	Epidemic	50%		96.4	50.7	93.2	90.8		81.5	68.3						
11259	CC8	Infection	Epidemic	100%	82.5	97.0		85.9	88.8		61.7	46.2	44.5					
11051	CC5	Infection	Sporadic	NA		84.7												
11086	CC8	Infection	Sporadic	0%		94.2												
11107	CC30	Infection	Sporadic	100%		95.6			42.9	82.5				93.3				25.4
11112	CC30	Infection	Sporadic	0%														
11122	CC22	Infection	Sporadic	50%		74.1												
11193	CC8	Infection	Sporadic	50%		93.7									71.4	61.0	20.3	
11210	CC5	Infection	Sporadic	0%		71.0												

Presence of bacterial virulence factors in NEM subnatants after inoculation with epidemic (n = 5) and sporadic (n = 7) MRSA as determined by a multiplex competitive Luminex assay. Passage through the keratinocyte cell layers and filter insert below was observed at 24 h, 0%: no passage; 50%: passage in a single NEM; 100%: passage in both NEMs. (1): Data are given as inverted (×−1) percentage of the untreated control; higher MFI values represent higher levels of virulence factors present. Values were considered relevant if the MFI values were >25% compared to the untreated control. Blanks indicate no relevant values. Presence of virulence genes is given as P: present, or A: absent.

### Passage of bacteria through the N/TERT epidermal models during infection

**Fig. 4.**
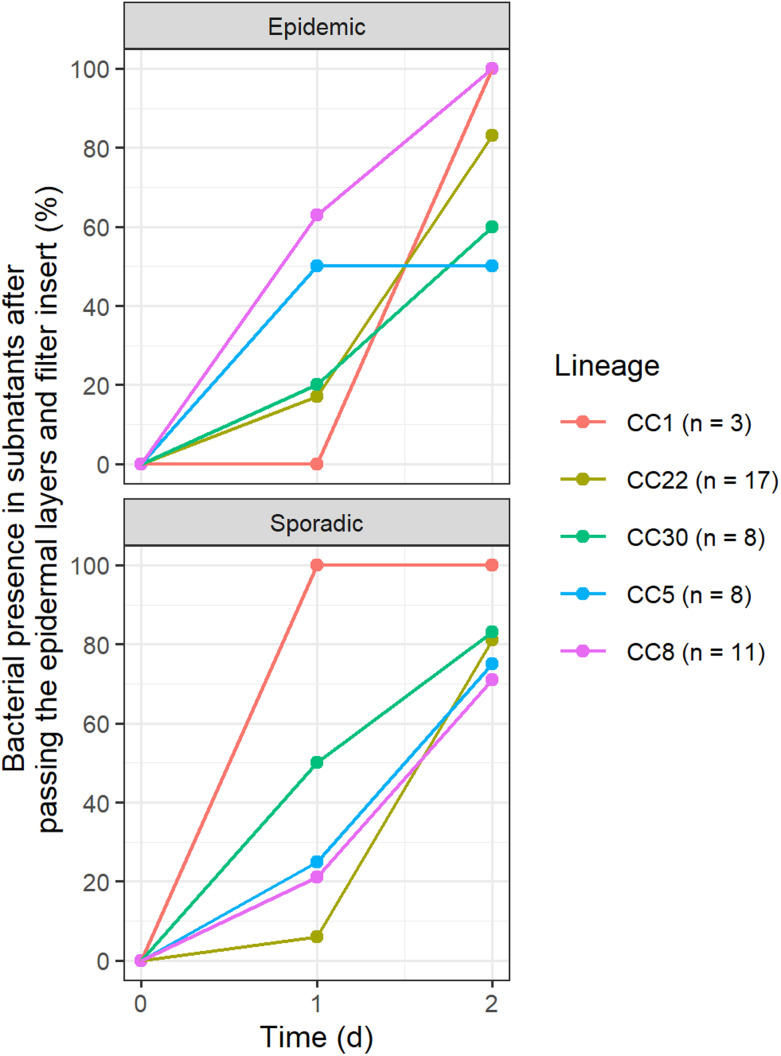
**MRSA within the culture subnatants upon interaction with NEM keratinocytes.** Bacterial presence in the subnatant indicate that bacteria have passed through the keratinocyte cell layers and filter below. Results are available for 27 epidemic and 31 sporadic strains and shown as percentage of models demonstrating penetration of epidermal layers. Subnatant bacterial presence increased from 27% at 24 h to 78% at 48 h and 100% at day 8.

We observed an occasional presence of bacteria in the culture medium indicating their passage through the keratinocyte cell layers and the filter insert below. A control experiment showed that strain LUH14616 did not penetrate the filter insert when keratinocytes were absent, indicating that their interaction facilitated passage. Bacteria were found in 27% of the model subnatants at 24 h, in 78% at 48 h and in 100% at 8 days. Subnatant contamination within 24 h was most frequent in epidemic strains of CC8 (63% of models, 3 of 4 strains) and CC5 (50%, 4/6) and in one sporadic CC1 strain (100%) as opposed to epidemic strains of CC22 (17%, 2/9) and CC30 (20%, 1/5) (Fig. 4). No association was evident between keratinocyte layer disruption at 24 h and the expression of the studied virulence factors.

## Discussion

This study explored the ability of 58 MRSA strains to survive on human epidermal models in relation to their epidemic or sporadic behaviour. Our data revealed a small but significant difference in bacterial counts between sporadic and epidemic strains on 3D epidermal models based on primary keratinocytes. Here, models exposed to sporadic strains showed lower bacterial counts than those exposed to epidemic strains. However, comparable counts were found for sporadic and epidemic strains on 3D N/TERT-based models. These data indicate that the outcome of interactions between MRSA strains and human skin may differ among donors. Potential factors leading to reduced skin colonisation by *S. aureus* are nutrient competition, production of antimicrobial peptides, sebaceous lipids and free fatty acids, and host-specific immunity [25, 26]. In this regard, we found significantly increased production of immune factors hBD-2 and IL-8 by NEMs after inoculation, which confirms the development of an innate immune response in our models upon colonisation by *S. aureus.* However, the expression of these immunological mediators by the models did not differ between sporadic and epidemic strains. Furthermore, interaction with N/TERT cells induced the expression of various pore-forming toxins, extracellular adherence proteins, immune evasion proteins and autolysins by epidemic strains primarily. Various studies have suggested increased skin colonisation by epidemic MRSA due to the expression of virulence factors [8, 27]. However, expression of toxins had no effect on bacterial survival in our study. In conclusion, these data suggest that both bacterial and human factors influence the complex interaction between pathogen and host and determine the fate of a *S. aureus* strain when exposed to human skin.

In our study, we found that epidemic, but not sporadic, MRSA strains expressed pore-forming toxins, including alpha-hemolysin (Hla), gamma-hemolysin (HlgB), PVL (LukS) and LukED, upon interaction with NEMs. Upregulation of *hla*, *hlgB*, *lukS-PV*, *LukF-PV*, *lukE* and *lukD* genes by *S. aureus* strains has been shown earlier in skin explants and in drainage material of cutaneous abscesses, which strengthens our findings [28–32]. All these toxins exhibit cytotoxic activity towards leucocytes, i.e., monocytes and neutrophils [33]. LukED and Hla are also cytotoxic to dendritic cells and keratinocytes, respectively [33, 34], which highlights the role of these toxins in bacterial invasion of the skin. Moreover, we observed passage of *S. aureus* through the cell culture filter, which was mediated by the interaction between the bacteria and keratinocytes. Most likely, factors produced by skin cells and/or bacterial cells facilitated the destruction of the filter. Passage of the filter was observed for all genetic lineages but occurred more quickly for epidemic strains of CC8 and CC5. Despite these observations, we were unable to show an association between keratinocyte damage and the expression of bacterial toxins.

Next to toxin production, we found expression of the major autolysin Atl, which plays a dominant role in peptidoglycan metabolism, and was therefore expected for all strains [35, 36]. Additionally, Atl contributes to surface attachment, biofilm formation and internalisation into host cells [35, 36]. Extracellular adherence protein (Eap) was also expressed. Besides its role in immune evasion, Eap affects the interaction between *S. aureus* and keratinocytes through interference with keratinocyte proliferation and migration, wound healing and stimulation of bacterial adherence and internalisation [37–40].

Earlier studies have shown higher expression levels of certain virulence factors in epidemic MRSA compared to sporadic MRSA. Higher expression of *hla* was found for USA300 compared to USA400 in a rat pneumonia model [7]. Hla expression was also higher in community-associated MRSA compared to hospital-associated MRSA [41]. Epidemic MRSA strain MW2 induced more cytotoxicity in human primary keratinocytes when Hlb was expressed [27]. Interestingly, despite our small sample size for virulence factor experiments, we found that toxin production was mostly limited to epidemic strains. Taken together, these results might imply that epidemic and sporadic strains are equally capable to survive on the epidermis, but epidemic strains might be more capable of responding to immune cells upon infection than sporadic strains.

To our knowledge, this is the first study that compares the ability of large numbers of clinical epidemic and sporadic MRSA strains of different genetic backgrounds to survive on human skin. It should be noted that the definition of epidemic and sporadic strains differed between originating countries due to different testing and sampling practices. This could have affected our results. As our models consisted of an epidermis only, another limitation was the absence of immune cells which normally reside in the dermis. For future work, skin models consisting of dermis and epidermis would be preferred to gain more insight into the innate immune response against natural MRSA infection.

Overall, we found that epidemic and sporadic MRSA strains were equally capable of surviving on epidermal models of the N/TERT cell line. Nevertheless, survival of epidemic strains was more successful than sporadic strains on epidermal models of primary keratinocytes, indicating a possible role for both host and bacterial factors in MRSA survival and transmission. Additionally, various pore-forming toxins, including alpha-hemolysin, PVL and LukED, were exclusively expressed by epidemic strains, giving these strains a benefit when encountering neutrophils. Together, these findings provide potential explanations for the transmission success of epidemic MRSA strains.

## Supplementary material

For supplementary material accompanying this paper visit http://doi.org/10.1017/S0950268822001765. click here to view supplementary material
